# Competing with the colonial scalpel: ‘Peaceful atoms’ in Cold War Taiwan

**DOI:** 10.1017/mdh.2026.10070

**Published:** 2026-10

**Authors:** Hsiu-Yun Wang

**Affiliations:** Department of Medical Humanities and Social Medicine, College of Medicine, National Cheng Kung University, Taiwan

**Keywords:** Hyperthyroidism, Radioactive iodine, Thyroidectomy, Nuclear medicine, Atoms for Peace, Colonial medicine, Cold War Taiwan, transnational history of science

## Abstract

This paper examines competing treatments—surgery and radioactive iodine (RAI, I131)—for hyperthyroidism in the context of post-colonial Cold War nuclear science in Taiwan. Although the U.S.-led Cold War Atoms for Peace project pushed for the globalization of nuclear medicine, it had to compete with the Japanese colonial legacy in Taiwan (Republic of China). This government-in-exile embraced nuclear science and ushered in RAI with staged rituals. However, this modern procedure failed to overtake the surgical approach (thyroidectomy); in fact, as late as the 1990s, well after RAI had become a standard procedure throughout the world, surgery was still the major treatment option for this common disease in Taiwan. The tension between the global and the local is rooted in infrastructure as well as the materiality, regulation and availability of the Iodine 131 isotope, and, finally, the customary practice of the surgical tradition, established since the colonial period. Thyroid diseases, especially endemic goiter, were an important issue in colonial medicine, and the study, diagnosis, and treatments of thyroid diseases were an integral part of colonial surgeons’ identity. Surgical clinics had been a fixture of institutional medicine throughout Taiwan by the mid-twentieth century. In contrast, with the exception of a few prestigious hospitals in Taipei such as the National Taiwan University Hospital, most medical institutions and clinicians were not equipped to use isotopes. The availability of I131 was also limited, as the nuclear reactor did not become active until the 1960s. The preference for surgery thus points to the significance of material and social conditions of medical practice in Taiwan, amid the backdrop of the haunting images of the atomic bombs dropped by the U.S. on nearby Japan which the Atoms for Peace Project had aimed to overcome.

On 24 October 1957, an inauguration ceremony for the first use of radioactive iodine-131 (RAI) was held at National Taiwan University Hospital (NTUH). During the event, the Director of the Atomic Council of Republic of China (Taiwan) and also the Minister of Education, Chi-Yun Chang (1901–1985), ‘dropped’ an iodine-131 capsule into a patient’s mouth. The fact that it was made into a public event attests to its perceived propaganda significance in ‘opening up a new era in the peaceful uses of atomic energy for Free China’.^2^ Imported from the U.S., it was reportedly the first use of RAI for treating hyperthyroidism in Taiwan.

However, a spectacular start is no guarantee of future success. Even though RAI had been introduced to Taiwan by the late 1950s, the dominant treatment for hyperthyroidism continued to be surgery when antithyroid medication failed to manage the condition or if a relapse occurred. No comprehensive historical statistics are available, but contemporary data provides a glimpse of the complexity of treatment options. In the U.S., radioactive iodine has been the preferred therapy since after the Second World War, with some exceptions such as children and pregnant women.^3^ In contrast, in Taiwan, among the new cases of hyperthyroidism (2000–2008), 99.68% received antithyroid drugs at the time of initial diagnosis, 3.51% of the cases received thyroidectomy in the first year of diagnosis, and less than 0.1% of the cases radioactive iodine.^4^ South Korea, another former Japanese colony, also received RAI (from the U.S.) about the same time; however, the paths taken in its use differed between the two countries.^5^ In Korea, according to a 2013 survey of members of the Korean Thyroid Association, 97.1% (133/137) reported choosing antithyroid drugs and the remaining 2.9% chose radioactive iodine as the initial treatment. Moreover, thyroidectomy has rarely been chosen for treatment in Korea.^6^ In contrast, during the first decade of the twenty-first century, there were still approximately 10,000 thyroidectomies being performed annually in Taiwan.^7^ Until 2015, in the words of Dr. Wei-Jen Yao, a nuclear medicine specialist, RAI was a ‘second line treatment’ for hyperthyroidism in Taiwan.^8^ Why did RAI in Taiwan assume this different trajectory?

Radioactive iodine, one of several isotopes that emerged in the context of the Atoms for Peace program, was both a global scientific endeavour and the U.S.’s way of maintaining its dominance in the postwar political order.^9^ As Angela Creager has pointed out, Atoms for Peace ‘marked a strategic policy shift from denying foreign nations nuclear materials and technologies to sharing these resources as a display of American beneficence and a means to secure diplomatic ties to the developing world’.^10^ Building on developing countries’ desire to develop nuclear technology, including the bomb, the U.S. created a market for nuclear reactors while limiting the use to ‘peaceful purposes’.^11^ It served as a ‘cultivation of desire’ for nuclear modernity in developing countries, as John Krige has observed.^12^

Given its geopolitical position and eagerness to catch up with the western world (mainly the U.S.), one might expect that RAI would have prevailed in Taiwan as yet another example of American global developmentalism. Yet, as recent studies on transnational history of science and technology have pointed out, border crossings pose many challenges, especially for a nation-state as tenuous and embattled as the Republic of China (Taiwan, ROC).^13^ RAI, first developed by the American physician Saul Hertz (1905–1950) in a thyroid clinic at Massachusetts General Hospital in the 1930s, is recognised as one of the most successful cases in the development of nuclear medicine by the U.S. after the Second World War.^14^ Nevertheless, little is known about what circumstances RAI encountered on the ground as the new practice travelled the globe to places such as the former Japanese colony of Taiwan.

This paper traces the history of the two competing treatments for hyperthyroidism in Taiwan since the 1950s – namely, surgery and RAI. I suggest not only that the treatments for a given disease are historically contingent but also that the globalisation of nuclear medicine is not a uniform and smooth process. In nuclear medicine, various diagnostic and treatment technologies are built upon different material and institutional conditions, and patients in different locales have their own particular perceptions regarding a specific diagnostic or treatment method based on how it entered the place. A new treatment for a certain disease has to compete with the existing treatments on at least the above-mentioned terms. These historical contingencies illustrate the uneven trajectory of globalisation as represented in the Atoms for Peace programs. By examining the local material conditions, medical traditions, and institutional settings in Taiwan at the time, I wish to elucidate and complicate the issue of how knowledge and practice move. Although RAI and thyroidectomy co-exist as treatment options today, the two developed in very different cultural, political, and material contexts in Taiwan. The preferred treatment for hyperthyroidism – thyroidectomy – is rooted in the colonial past when surgeons were engaged in the study and treatment of goitre, a disease caused by iodine deficiency. To be sure, hyperthyroidism and goitre are distinct diseases with different aetiologies,^15^ but they share certain symptoms and, until antithyroid medications were available in the 1940s, treatment by surgery. In what follows, I first trace the history of the existing treatment, thyroidectomy, to its colonial past, then I examine how radioactive iodine entered the scene, particularly its material condition or constraints. Only by bringing the history of surgery into the story can we comprehend the complexity of the globalisation of nuclear medicine.

This study is anchored in and contributes to recent scholarship emphasising transnational approaches in historical analysis. Specifically, it builds upon research that highlights the interwoven nature of local developments with global geopolitical and medical currents. Yu-Ling Huang’s case study analyzes the production of biopolitical knowledge in the complex dynamics of various factors – Cold War and Civil politics, Taiwanese and U.S. actors, and fertility studies and the data generated by fertility studies.^16^ Honghong Tinn’s analysis of Taiwan’s computing industry, though not about medicine, demonstrates how Cold War geopolitics – marked by the synergy between U.S. dominance and the nationalist government’s nation-building initiatives – fundamentally structured industrial development.^17^ This geopolitical context also significantly informed medical practices, including hyperthyroid treatments, which were integral to state and security concerns of the era. Wayne Soon’s work on the history of biomedicine in China highlights the critical role of overseas Chinese figures, such as Lim Boon Keng, Robert Lim, and Wu Lien-Teh. His research underscores the perennial interconnectedness of local and global dynamics in the evolution of modern medicine and public health.^18^

Extending this focus, Harry Wu’s investigation into the World Health Organisation’s (WHO) quest for a global standard in mental health illuminates the fundamental importance of transnational medicine in establishing global health norms.^19^ Both Wu’s book and this paper are concerned with the contested nature of the globalisation of science, whether in psychiatry or nuclear medicine. While Wu identifies the ‘negotiation’ between universal psychiatric metrics and local variations, this paper demonstrates that surgery was a formidable competitor vis-à-vis the U.S.-backed RAI. Though the configurations of power are different (WHO vs. U.S. hegemony), the position of Taiwan is similar in both cases – as an ‘ideal bedfellow’ and an ‘international laboratory’ in the case of the WHO’s global project and, in the case of nuclear science, as a geopolitically important client state. However, how ‘colonial legacy’ functioned in the two is strikingly different. While postwar psychiatric research in Taiwan was made possible by utilising the household registration and hokō system (or bao jia) established by the Japanese colonial administration, the surgical tradition established during the colonial period served as a persistent rival to RAI.

Furthermore, Shinyi Hsieh’s dissertation on the Naval Medical Research Unit No. 2 (NAMRU-II) reveals the complex interplay of historical legacies and contemporary power dynamics. By incorporating the perspectives of marginalised groups, Hsieh’s work demonstrates how the enduring impact of the Japanese colonial legacy, the priorities of the Taiwanese nationalist agenda, and the exercise of U.S. domination collectively contributed to a logic of coloniality in the post-war period.^20^

## Surgery: A colonial legacy

Before 1960, except for those who had come with the KMT army (1,194) and been granted a license regardless of their medical education status, most medical practitioners were trained during the colonial period within the Japanese Empire, including Taihoku (Taipei), Japan, and Manchuria.^21^ This body of medical practitioners and their practices, along with other elements of the infrastructure established by the Japanese colonial government – including the Medical College of Taihoku Imperial University and public hospitals – constituted a colonial legacy which continued to play a significant role in medicine after the war. In the case of goitre, the two surgical departments at NTUH were particularly foundational.

Thyroidectomy had been a treatment for both goitre and hyperthyroidism since the early twentieth century, and, as in other parts of the world, goitre fell within the domain of surgery in colonial Taiwan. Goitre was one of the major endemic diseases that colonial officers identified as early as 1902, when *Journal of Formosan Medical Association* (hereafter, *JFMA*), Taiwan’s flagship medical journal since the colonial period, was first launched. Tsugio Horiuchi, an appointed faculty at Taipei Medical School (after 1936 Faculty of Medicine of the Taihoku Imperial University, currently College of Medicine, National Taiwan University), published a survey of endemic disease in the first volume of *JFMA*, in which goitre ranked third.^22^ Tomoe Takagi (1858–1943), principle of the Taipei Medical School and founder of *JFMA*, also noted goitre in his *Die Hygieneschen Verhältnisse der Insel Formosa* (Hygiene Report on Formosa) in 1911.^23^

Indeed, goitre attracted considerable attention from epidemiologists, pathologists, and surgeons who carried out field surveys, explored aetiologies, and tested surgical treatments – all of which were discussed in *JFMA.* In the period between 1902 and 1945, the *JFMA* carried 112 articles, speeches, and reports on thyroid diseases, particularly goitre. The research methods, questions, and scope were diverse, including goitre’s geographical distribution; rates among different age, racial, and vocational groups (newborn, school children, aborigines, and fishermen); healing after surgery; metabolism; prevention and treatment; iodised salt as preventive measure; increase during the war; drinking water; iodine contents in food stuff; and aetiology. Colonial medical researchers also experimented with iodised salt for school children as a preventive measure.^24^ In short, the goitre research as seen in the *JFMA* was a field in which large-scale public health surveys and experiments, hospital-based clinical research, and laboratory-based experiments co-existed.

Most of the research relied on the colonial state administration and Taipei Medical School/Medical Faculty of Taihoku Imperial University which enabled researchers to organise and collect specimens and data from different regions and schools. Members of the two surgical divisions at Taihoku Imperial University Hospital, Heijirou Sawada’s First Surgical Department and Kunio Kawaishi’s (1895–1972) Second Surgical Department,^25^ carried out the majority of the research. According to Kawaishi’s disciple, Yoshio Hashimoto, Kawaishi’s research on goitre employed a total of 318,116 investigators and found 26,979 patients, including 19,974 out-patients and in-patients.^26^ Additionally, thyroid diseases were also a common topic for degree-granting research.^27^

The interest in goitre was an integral part of colonial medicine, and as Yen-Chiou Fan has pointed out, in the late 1930s Taiwan was the Japanese Empire’s key experimental site for goitre prevention and treatment.^28^ The researchers saw taking on the goitre problem as a matter of import to imperial expansion, and, since ‘Taiwan is the only place within the nation [Japanese empire] where endemic goitre spreads’, the knowledge and solutions to be gained in Taiwan were instrumental.^29^ Kawaishi writes that the issue of goitre:

…has great significance from both a medical standpoint and in colonial policy, especially during the time when Japan is planning to advance southward into the continent, including southern China, India, and the Dutch East Indies.^30^

Members of Kawaishi’s lab reiterated this point when they published the aforementioned article on iodised salt as a prevention method.^31^ As colonial physicians, they were competitively sensitive to what other physicians and other countries were doing in the world. For instance, they paid attention to the different iodine dosage recommendations by Swiss physicians Heinrich Hunziker and O. Bayard, and they were also aware of iodised salt being used in the U.S.

A further look at other studies on goitre carried out by officers working in government prefectural hospitals reveals a different kind of relationship between goitre and colonialism. For example, Fujibayashi Kenji’s ‘Endemic and Experimental Aspects of Goitre’ was a commissioned report by the Local Disease Investigation Committee. The investigation focused on areas like Mata’an, Tapalong, and Pattsu in Hualien. In addition to Fujibayashi noting the difficulty in acquiring precise facts due to the short timeframe and the fact that most patients were aborigines who often avoided hospitalisation or fled investigators, he observed that goitre ‘seems to exist in large numbers in indigenous areas not yet subjugated’.^32^ Whereas Kawaishi framed the importance of goitre in terms of imperial expansion, Fujibayashi saw it as an issue of assimilation.

The problem of goitre varied within the Japanese Empire. Physician researchers responded differently to the issue of goitre according to their locality. Goitre in Korea was a ‘comparative rarity’, and could only be found in the Kangai area in the northern hilly section of the country. Similarly, goitre was considered rare in Japan; it was ‘not a problem in any magnitude’, according to the World Health Organisation.^33^ However, in an attempt to repudiate the notion that there was no goitre in Japan, Shigenori Takenaka (1864–1925), a former military physician who practiced in Tokyo, pointed out that there were indeed cases found in Hokkaido in 1914. He reasoned that the rarity was in part due to the fact that ‘patients’ did not see goitre as a disease.^34^ Nevertheless when Kawaishi and Hashimoto published their article with the title of ‘Endemic Goitre in Japan’, which focused on territories outside of ‘Japan proper’, they briefly repudiated J. F. McClendon’s assertion that Honshu and Hokkaido were goitrous areas as only five cases were found among a population of 2,500. They provided a summary of the distribution of goitre in Japan proper and then devoted the larger part of the article to the situation in Formosa (Taiwan).^35^

In contrast to ‘Japan proper’, goitre was a much more severe problem in Manchuria and Taiwan. During the first few decades of the twentieth century, several studies were carried out by Japanese officers in Manchuria. In these articles one does not find the imperial expansion rhetoric expressed by Kawaishi,^36^ but apparently it was a threat to Japan’s empire-building. For instance, Takei and Takamori noted that, in addition to the rate being between 10% and 85%, immigrant Japanese rapidly became victims.^37^ The study of goitre was also at the centre of colonial medicine in Manchuria, as manifested in the infamous case of interviewing, dissecting, and colleting body parts of captured Manchurian resisters by Hisao Kubo, a professor at the Mudken Medical College.^38^

In clinical practice, both Kawaishi and Sawada, the two leading surgeons at Taihoku Imperial University, were not just researchers of the disease. They were also surgeons known for their skills in thyroidectomy,^39^ which was an essential part of surgical training. Tian-Yu Lin (1913–1995), a prominent surgeon at National Taiwan University Hospital, remembered the thrill of doing thyroidectomy as an intern during the 1930s.^40^ The two colonial surgeons trained Taiwanese disciples such as Lin in goitre research as well as surgery. Those trainees made up the bulk of the surgical practitioners after the war.^41^

**Figure 1.**
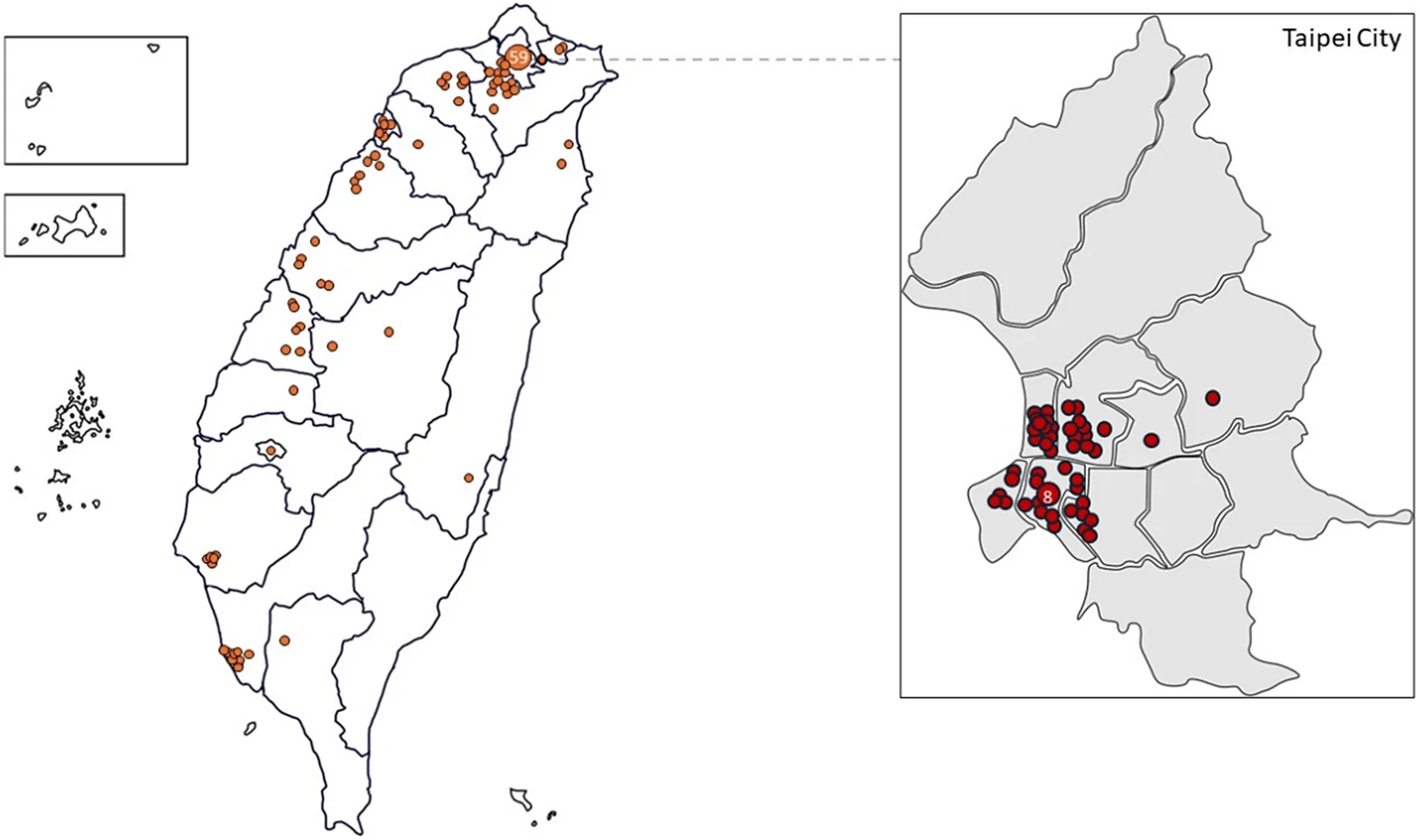
Tien-Cheng Kao’s network of surgical clinics in Taiwan, 1959.^45^ Figure 1. long description.

Outside of the Imperial University, in terms of clinical practice, before the 1930s, surgery was limited to hospitals that were equipped with surgical resources, and private surgical clinics were few and most were run by Japanese. However, by 1959, when Tien-Cheng Kao,^42^ the superintendent of NTUH and a surgeon, celebrated his tenth anniversary of working at the Hospital, a list of 135 surgeons and anaesthetists were printed in the event brochure, probably to show that the field of surgery had made considerable progress. It is also an indication that surgical clinics had become a common place in Taiwan (Fig 1).^43^ It is in this context that Cong-Ming Tu (1893–1986), one of the most prominent medical men in post war Taiwan, praised Kao for having the foresight to choose to go into surgery after graduating from the School of Medicine, University of Tokyo (1930). Very few people had dared to specialise in surgery at the time of Taipei Medical School (or Taiwan Sotokufu Igakko, later NTU Medical College) during the 1920s and 1930s, according to Tu.^44^

As endemic goitre was a serious issue, surgeons had abundant opportunity to do thyroidectomy.^46^ Surgery was considered safe by the 1940s.^47^ According to the medical records of *Xu Waike* [Xu’s Surgical Hospital], a local hospital established in Kaohsiung in 1956, surgeries totalled 1,044 between August 1958 and July 1959; thyroidectomies took third place (127 cases), after hysterectomies (138) and appendectomies (248).^48^ It comes as no surprise that thyroid disorders and thyroidectomies were familiar topics in gentries’ diaries.^49^ Pong-Xing Xu (1909–1984), the founder of *Xu Waike*, was also one of Sawada’s disciples who had studied goitre.

Some surgeons even made their name by the sheer number of thyroidectomies they accomplished. Take Harry Miller of the Seventh Day Adventist Hospital (later Taiwan Adventist Hospital) as an example. A news report in 1957, the year when iodine-131 was introduced to Taiwan, marvelled at his achievement: ‘During the 1920s, traveling around in Asia, he performed 6 to 8 thyroidectomies daily. He has done about 3000 surgeries so far…’.^50^ According to a biography of Miller, he successfully reduced the mortality rate after surgery from 50% to 1%, and throughout his life he performed a total of 6000 thyroidectomies.^51^ The well-known Chinese poet and scholar Yu-Ching Sheng (1893–1965) was one among those thousands of surgical cases. Her essay detailing her illness experience, of seeking treatment from numerous physicians in vain, and gaining eventual relief through surgery in 1935, was a powerful testimony of the triumph of surgery over traditional Chinese medicine – earning Miller the moniker ‘thyroid Miller’.^52^

Due in part to the reduced mortality rate and the flourishing of surgical clinics after the 1950s, by the 1970s thyroidectomy had become one among several surgeries performed to excess in Taiwan (the others were hysterectomy, stomach removal, and appendectomy).^53^ Additionally, the majority of the patients who suffered from hyperthyroidism were women. Several newspapers reported six or seven out of ten women factory workers had a surgical scar on their neck, and there was even a popular saying that went with the phenomenon: ‘Women labourers in Taoyuan lose three-fourths of their thyroid and some people in central Taiwan have lost two-thirds of their stomach’.^54^ Not surprisingly, these reports drew public attention, including from writers, medical practitioners, and politicians. A Legislative Yuan member, Pei-Lan Wei (1911–1986) raised the question of surgical abuse: ‘Certain medical practitioners, in order to make money from labour insurance, have performed unnecessary thyroidectomy on women labourers’.^55^

Tai-An Zheng, a psychiatrist, cautioned that some doctors ‘misdiagnosed’ or ‘intentionally misdiagnosed’ patients, and treated cases of anxiety disorder as thyrotoxicosis (i.e., high levels of circulating thyroid hormones, also known as thyroid storm). ‘They even cut off patients’ thyroids…. Whoever has the above symptoms should go to a reliable physician or hospital for detailed and correct examination’.^56^

Surgeons in elite hospitals also publicly sounded the alarm about surgical clinics outside of Taipei engaging in suspicious conduct. Dr. Kaung-Yi Liao, a NTUH surgeon, was quoted as saying that although ‘thyroid tumours may be malignant or benign, not all require surgery’, and ‘whenever they [hospitals in central and southern Taiwan] see patients with thyroid tumour, they all use the knife’.^57^ Feng Li, an NTUH pathologist, advocated instituting pathology examinations to prevent surgical abuse.^58^ By the 1990s, there were even rumours of ‘thyroid-less villages’ along with rumours of ‘uterus-less villages’.^59^ There are certainly differences between hysterectomy and thyroidectomy, yet uterus and thyroid were allegedly among the most frequently removed organs. In the meantime, likely as an attempt to counter the trend and to prove their professional integrity, the Taiwan Surgical Association and the Endocrine Society of the Republic of China published a *White Paper on the Thyroid*, which recommended the use of antithyroid medication first and surgery only if a physician’s evaluation determines it to be necessary.^60^ It is worth noting that one of the surgical abuses, hysterectomy, was also a global occurrence. It went with the notion of ‘the useless and pathological uterus’ (after a woman has given birth to ‘enough’ children), first advocated by the American ob/gyn Robert C. Wright in the 1950s; by the 1970s, various versions of this assertion could also be found in East Asia, including Taiwan and Japan. It was a convenient justification for a prophylactic hysterectomy. In the case of Taiwan, family planning, cancer prevention campaigns, and surgical tradition provided fertile ground for it to flourish.^61^

## Cold War science and iodine-131

Cold War Taiwan was a postcolonial island where the Republic of China, a state in exile, relocated.^62^ In this context, nation-building consisted of re-taking China (as in the phrase fangong jianguo, anti-Communism and building the nation), asserting its legitimacy as the authentic China, and strengthening national prowess. Science and technology stood at the centre of this kexue jianguo (science builds nation) project.

Despite being promoted in the name of peace, ‘Atoms for Peace’ provided an opportunity to advance nuclear reactor technology for scientific and potential military applications, even though it was ostensibly limited to peaceful purposes. The content of nation-building included ‘restoring’ National Tsing Huan University, building a research nuclear reactor and establishing laboratories at NTU Hospital, the Veterans’ Hospital, and other research institutions. RAI, presented as a novel scientific cure, was a model item in the Atoms for Peace project that ‘Free China’ could apply to ‘bomb’ the disease of hyperthyroidism and thereby claim prowess and achieve ‘catching up’ with the West.

At the ideological level, U.S.-sponsored nuclear science was a symbol of peaceful democratic progress and anti-Communist propaganda. Government officials such as Chi-Yun Chang, the first chairman of the Atomic Energy Council of Taiwan, welcomed the ‘Atomic Age’ that was ‘this new [nuclear science] outlook on the world and on life that has the effect of completely eradicating the materialism on which Communism is based’^63^; he stated that ‘the development of the peaceful uses of atomic energy will spell the doom of Communism….’^64^ This view reflects the ‘naïve assumptions about the incompatibility between science and Communism’ that some scholars have pointed out.^65^

Soon after Dwight D. Eisenhower gave his ‘Atoms for Peace’ address before the General Assembly of the United Nations in 1953, the United States began to promote isotopes in many developing countries in the name of peaceful applications of nuclear energy. The signing of the agreement for cooperation ‘Concerning Civil Uses of Atomic Energy’ with the U.S. government in 1955^66^ opened the atomic age in Taiwan. The first shipment of radioisotopes arrived that year, and though it was not indicated which isotopes were shipped, the historical records suggest it included iodine-131. A series of projects were started, including the aforementioned ‘restoration’ of National Tsing Hua University and the building of a research nuclear reactor there, the opening of several isotope labs, the sending of scientists and technicians to the West – mainly the U.S. – for training, and the arrival of experts, equipment, and isotopes from the U.S.^67^

As the local elite institution, National Taiwan University was the first institution to take part in applying radioisotopes, mainly iodine-131, in research of thyroid. When NTUH established an isotope research lab in 1957, they expressed their latecomer’s ambition of catching up with the U.S.: ‘There are one-thousand hospitals in the U.S. that are equipped with radioactive iodine treatment. In the future, when National Taiwan University Hospital can purchase radioactive isotopes, Iodine-131 and Cobalt-60 will be our priority’.^68^

To be sure, the Faculty of Medicine of NTU was not the only medical school in Taiwan; another one – National Defence Medical Centre (NDMC; currently National Defence Medical University) – had been relocated from Shanghai to Taipei in 1949.^69^ It is worth noting NDMC’s role in this history when, in the 1950s, medical applications of nuclear science were being introduced under the broader project of Atoms for Peace program. Chang Chi-Yun’s Taiwan AEC report explicitly refers to NTU and NTHU as the primary target institutions for Atoms for Peace; when mentioning the Ministry of Defence, ‘research institutions’ is alluded to without naming the NDMC. There are two possible reasons for this. First, from the standpoint of the U.S. AEC, the main organisation in charge of the Atoms for Peace program, which emphasised its non-military use, the NDMC was not a civilian institution.^70^ Supporting this view is the fact that the U.S. military, when evaluating whether to relocate NAMRU-II to Taiwan, considered NDMC as a representative of the military.^71^ Even though both NMDC and NTUH received American aid during the 1950s, when NTHU was constructing Cobalt-60 unit and isotope laboratory along with other infrastructure, NMDC was constructing students’ dormitory and the laboratories for pharmacy and gross anatomy.^72^

Second, different medical technologies appeared to have taken different trajectories historically. A Department of Medical Physics was established soon after NDMC’s relocation, led by Jing Wu (1905–1991), a radiologist who was trained under Paul C. Hodges (1893–1997) at Peking Union Medical College. Both Hodges and Wu were X-ray diagnosticians. The importance of X-rays was closely tied to the prevalence of tuberculosis.^73^ Wu had been a prominent radiologist in China, and he continued his work after moving to Taiwan in 1948. Wu frequently wrote articles promoting atomic medicine (yuanzi yixue) in the popular press, and Tien-Cheng Kao praised him as ‘a pioneer of atomic medicine in free China’.^74^ Radiologists’ use of X-way machines differed from the application of radioiodine 131 but was similar to the use of Cobalt-60 as the latter also involved image-making machinery.^75^

Wu attempted to develop atomic medicine but did not succeed.^76^ He served as a delegate to two International Atomic Energy for Peaceful Purposes Conferences (1955, 1958) and founded the Yuanzin Yixue Yanjiu Yuan (Research Institute for Atomic Medicine) under Executive Yuan in 1956, which soon purchased a cobalt-60 machine. Wu Jing subsequently left Taiwan in 1959 to accept a visiting professorship at the University of Southern California and never returned, and he would continue his work in radiology abroad. Perhaps in part because he was rarely in Taiwan during this period,^77^ the Yuanzin Yixue Yanjiu Yuan was disbanded that same year, and the cobalt-60 machine was transferred to Chung Hsing Hospital (currently Taipei City Hospital Chung Hsing Branch).^78^

The forward-looking enthusiasm for isotopes expressed by Dr. Tien-Chen Kao (1904–1964), the superintendent of NTUH, is apparent in his various transnational exchanges and writings. In the years following 1955, Kao was engrossed in various nuclear medicine-related activities, including the visit of Marshall Brucer (Director of Oak Ridge Institute of Nuclear Medicine) and members of the American Atomic Energy Commission (U.S. AEC), the ground-breaking of the Radio-Isotope Lab at NTU Hospital,^79^ and the first use of iodine-131 on patients with hyperthyroidism. In 1958, when a cobalt-60 radiotherapy/teletherapy machine was installed in NTUH, Kao presented a paper on the study of iodine-131 thyroid uptake at the second International Conference on Peaceful Uses of Atomic Energy in Geneva,^80^ which was subsequently submitted and published in 1960.^81^ Additionally, he published an article entitled ‘Clinical Use of Radio-isotopes in Surgery’^82^ in *JFMA.* In 1959, he celebrated his 10th year anniversary at NTUH. At the ceremony, he noted the newly completed cobalt-60 radiotherapy/teletherapy machine room and the radioisotope room^83^ among his major achievements.^84^ In the midst of his research and numerous official activities, he also authored several popular magazine essays on the promises of nuclear medicine.^85^

It is not surprising that in this context Kao framed the newly arrived isotopes in the tradition of colonial medicine. He writes,

In the past, the two surgical departments headed by Sawada and Kawashi had already achieved considerable success. One of their common research areas was endemic goitre. Due to equipment limitations, our surgical department temporarily suspended research on this topic. In October 1957, our hospital’s radioisotope therapy room began using radioactive iodine, and we started preparations to study this endemic goitre.^86^

In the popular media, promoters of iodine-131, including Kao and his colleagues at NTU Hospital, trumpeted iodine-131 over surgery.

Many men and women in Taiwan suffer from hyperthyroidism. In the past, surgical removal of the thyroid was a must…. Hsueh-Fang Yang, Tien-Cheng Kao, Kuan-Chu Wang, Yao Chang-Chien, Chao-Ming Ho, and Chen Ruei-San of the National Taiwan University Isotope Research Institute gave a paper today at the [Taiwan Medical Association] annual meeting, and they explained that the application of radioactive iodine as a treatment for hyperthyroidism. This would make surgery unnecessary.^87^

In addition, not only were the effects of iodine-131 praised, but they were even described in magical terms:

One who takes iodine 131 as if drinking a glass of water will receive its novel effects, of iodine releasing its energy among the clusters of atoms, thereby slowing down the [body’s/patient’s] metabolism. Yet how it works of it is still unclear.^88^

Dr. Jing Wu (1905-1991) also expressed the same kind of enthusiasm. In the Atomic Age, he writes, ‘radiation has claimed [mastery] over the diseases that cannot be cured by medications and surgery’.^89^ In short, the promoters of RAI enthusiastically saw it as a replacement for surgery.

This enthusiasm in the reception of RAI was striking, especially compared to prior to its arrival. Just one year before the first use of RAI, Xue-Fang Yang, an internist at NTU Hospital, evaluated the three different treatment options for hyperthyroidism in a *JFMA* article:

This radioactive iodine treatment is simple to administer, does not hinder patients’ daily life, and is extremely convenient. However, radiotherapy requires expensive equipment and skilled experts who can handle radioactive materials. According to the current situation in Taiwan, the possibility of its implementation is very low.… The recent development of anaesthesia and the emergence of antithyroid drugs have greatly reduced the mortality rate after surgery.^90^

He went on to say that, based on his experience since 1953, the use of antithyroid medication required patience from both the patient and the doctor. It had the additional disadvantages of being expensive in Taiwan and having a high recurrence rate. Even though he qualified his conclusion with the assertion that all three treatments were not ideal, it reads as though surgery was the better choice than the other two. Coming from an internist, this assessment was rather convincing.

However, soon after RAI was introduced, Yang changed his position. RAI now was the best treatment. In a paper authored by a group of physicians at NTUH, including Yang, one finds an approving view of RAI: ‘Not until 1941 did Hertz et al and Hamilton et al begin to use iodine-131 on the patients with hyperthyroidism. Since then, it has been widely used on hyperthyroidism all over the world. And after much discussion, it was still chosen as the best treatment’.^91^

Elite physicians were confident that RAI would replace surgery and antithyroid medication. Chuan-Ching Chen, a radiologist at NTU Hospital, reported that in the period between 1959 and 1961, the number of cases using RAI as a treatment was 348. Dr. Chen was confident that RAI ‘has replaced surgery and the drug used in internal medicine’.^92^ In 1969, according to a newspaper article, National Taiwan University Hospital allegedly treated 815 cases of patients with hyperthyroidism using RAI, and they claimed that the cure was over 80%, except for a few who were resistant to RAI and were then ‘persuaded to accept surgery’.^93^ Occasionally, one could find certain ob-gyns who also supported RAI.^94^ It was also used for thyroid diagnostics, but discussions about the diagnostic applications seemed to be limited to physicians and rarely occurred in public settings.

It might seem unreasonable that some surgeons would promote a treatment that would render surgery unnecessary. In the post-war era, as mentioned earlier, Kao placed the introduction of the newly arrived isotope iodine-131 within the surgical tradition of Taiwan’s colonial past. The use of RAI for research was, in his words, one of the greatest examples of a ‘contribution to the field of surgery in the world’, and the fact that RAI was being used in a study of 88 goitre patients in Liu-Chia Township, Hsin-Chu County was a sign that NTU Hospital was catching up with the progress of science.^95^ In other words, in promoting RAI as a sure sign of scientific progress (a view advanced by Taiwan’s hegemonic patron, the U.S.), Kao also paid a tribute to the recent past of colonial medicine.^96^ It is revealing that the aforementioned brochure that was distributed at Kao’s 10th Anniversary contained the above assertion as well as a list of 135 surgeons all over Taiwan: iodine-131 sits in the middle of the colonial legacy – the surgeons’ tradition.

At the same time, advocates of nuclear medicine were aware of the Cold War climate and the urgency of nation-building vis-à-vis the threat of China to the ROC’s legitimacy and sovereignty. All things ‘nuclear’ were associated with ‘Free China’.

## The materiality of iodine-131

Indeed, radioisotopes had many advocates – the U.S. AEC, IAEA (International Atomic Energy Agency), the developmental state of Taiwan, American scientists, and elite medical scientists in Taiwan. IAEA promoted radioisotopes as being beneficial to developing countries, for ‘they were cheap, required small staff, and the technique is simple’.^97^ This was not the case for iodine-131 in Taiwan, however. While its mark on the few elite hospitals such as NTU Hospital was striking, it could hardly compete with surgery probably even within Taipei. It faced various challenges – including the lack of availability, its high cost, and its position within a professional niche.

RAI *was* costly. The chemical itself was expensive, and, since it had to be air-transported from the U.S., the total cost was beyond the reach of most patients. Each capsule was 200 New Taiwan Dollars (NT), compared to 3.08 NT for one kilogram of rice. In other words, one treatment was the equivalent of two-thirds of a person’s yearly consumption of rice (98–100 kilograms of rice).^98^ When the first six patients received iodine-131, the cost was split between NTUH (100 NT) and the patient (100 NT).^99^ A patient might need up to three treatments of RAI. The cost of thyroidectomy during the 1950s was probably somewhere between 100 and 150 NT.^100^ In the period between 1960 and 1977, according to Labour Insurance’s ‘Diagnosis and Treatment Payment Standards’, removal of goitre was 630 NT and Basedow thyroidectomy was 1350 NT. However, it is revealing that RAI was not included in the Labour Insurance’s ‘Diagnosis and Treatment Payment Standards’ in Taiwan until 1990, and even then only nine medical centres were qualified to offer RAI. In contrast, thyroidectomy (surgical removal of the thyroid gland) was included at least since 1960, just 4 years after Labour Insurance began to pay for medical services.^101^

Due to its relatively short half-life (8 days, especially when it had to be transported from the U.S.), the hospital would order iodine-131 from the U.S. after 10 patients had registered for the treatment,^102^ which were additional administrative, handling and scheduling burdens to using it.

The half-life of radioactive iodine is only 8 days, which poses a challenge for storage. It is ordered on demand; it will be air transported from the U.S. upon request…. [T]he cost for air transport is especially high. The eight pills arrived last Friday and after today they will lose radioactivity…^103^

RAI would have been more accessible if it had been locally produced in sufficient quantities. Even though the first nuclear reactor at Tsing Hua University became active in 1961 (THOR, a swimming-pool reactor), domestically produced Iodine-131 did not become available until 1964. Even then, it was initially available only to a few institutions – NTUH, NTU College of Agriculture, the Veteran’s Hospital and the American Naval Medical Research Unit 2 (NAMRU-II). Chien-Chang Li, the technician in charge of the RAI production, had difficulties producing it in large quantities during the 1960s. ‘At that time [1962], because the equipment was rudimentary, the manufacturing procedures were only small-scale in the laboratory. However, after the director announced the success of the development, what followed was a big order, and they needed a thousand times the amount that my lab could produce in micro-scale’. Attempting to meet the order amount, Lin had to stretch its production and ended up causing a contamination.^104^

In the period between 1964 and 1987, its production went through a series of improvements, including completion of a ‘new factory’ in 1966, conversion to a second generation of wet method in 1979,^105^ and establishment of a third-generation wet production method in 1984. It was claimed that the quality of RAI production reached its highest in 1987.^106^ In spite of the existence of this domestic production, the source of radioactive iodine for some physicians’ research in the 1970s was from Japan instead of THOR.^107^ In short, the domestically produced RAI did not meet local needs in terms of quantity and quality.

The adoption of RAI in Taiwan faced obstacles beyond mere lack of availability, as the local institutional setting was inadequately prepared for nuclear medicine. Although the first recorded use of RAI and the establishment of the NTUH’s Isotope Lab in 1957 marked early ceremonial beginnings, institutionalisation remained slow. The newly established Taipei Veterans General Hospital (VGH), associated with the NDMC, also set up a radioactive iodine lab and a scanning lab during the late 1950s. However, VGH did not establish a dedicated isotope unit ( *ke* or department) until 1966, initially placing it within the Radiology Department, which was focused primarily on X-rays; this unit finally achieved independent status as the Department of Nuclear Medicine in 1974. The institutional focus during this period remained heavily on radiology^108^; for example, shortly after NTUH purchased a Cobalt-60 machine in 1958, VGH’s Radiology Department acquired one in 1960, and its prioritisation of traditional radiology was further indicated by its invitation to Paul C. Hodges and his colleagues from the University of Chicago to provide assistance for two years during the 1960s. Consequently, the dedicated isotope division (which became the Department of Nuclear Medicine) was established much later in 1966, with Chih-Teh Loo (1901–1979) recruiting Shin-Hwa Yeh (1928–2017) in 1967, an NDMC alumnus at Stanford University, as its first director, meaning it took nearly two decades for isotopes to secure a stable institutional niche. Furthermore, institutionalisation was not only slow but also sometimes impermanent, as illustrated by the short lifespan of the aforementioned Yuanzi Yixue Yanjiu Yuan.^109^

The field of nuclear medicine^110^ did not seem a promising one for young medical graduates in Taiwan; in the words of Dr. Wei-Jen Yao (b.1952–), the practitioners ‘didn’t operate, didn’t prescribe drugs, and could not do private practice’.^111^ In short, it was not a lucrative path. The early practitioners in nuclear medicine were mostly graduates of NDMC. As enrolment in the college was considered military service, they paid no tuition. They were often made to enter certain fields based on national policy, and nuclear medicine was an example of this policy. For instance, Dr. Yao, as the top student in his class, was presented with two choices – go to the U.S. to earn a PhD in nuclear medicine on scholarship or serve in the military as a physician. Dr. Yao chose the former. When the U.S. severed official relations with Taiwan in 1979, he was no longer able to go. Nonetheless, he did choose nuclear medicine as his specialty.

There were also conflicts and tensions among professional societies. The Radiological Society of the Republic of China (currently Taiwan Radiological Society) was established in 1951, and its early leaders were radiologists trained in China before the war whose tool of the trade was the X-ray machine. As discussed earlier, Jing Wu, who was trained at Peking Union Medical College under Paul C. Hodges, was remembered as the go-to authority whenever someone needed an X-Ray diagnosis, whether a commoner or a dignitary.^112^ Its official journal did not exist until 1976. The Taiwan Nuclear Medicine Society did not come into existence until 1988, two decades after the expatriate nuclear medicine expert Dr. Shin-Hwa Yeh took an offer at the Veterans General Hospital in 1967.^113^

When practitioners in nuclear medicine wrote about the history of nuclear medicine or radiology, machines featured prominently in their stories – X-rays, cobalt-60, and caesium-137; in contrast, despite the fact that the measurement of thyroid did involve some equipment,^114^ these origin stories have never included accounts of RAI.^115^ Given the ongoing importance of these machines (such as cobalt-60 teletherapy machines) in cancer treatment, it is understandable that they took centre stage. These machines were expensive, but they conferred power on their users.^116^ The application of RAI for the treatment of hyperthyroidism did not involve any machinery and, unlike the beloved machines, it did not play a significant role in practitioners’ professional identity.^117^

Coming from the U.S., RAI was a commodity and practice exclusive to elite practitioners such as Kao and Wang in the top medical facilities. Before the 1980s, whether in academic journals or popular media, the presence of RAI was limited to elites’ writings, for the most part by NTU-affiliated researchers and practitioners. By comparison, local practitioners viewed atomic medicine as a novelty cure. A newspaper medical advertisement by Yan Neike Yiyuan (Yan’s Hospital for Internal Medicine) in Tainan city, promoted yuanzineng dian (atomic iodine) as one of its specialty items. The ad claimed that ‘take it one time internally and goitre is cured’. Dr. Yan, the superintendent, was a liumei boshi (a person with a PhD from the U.S.).^118^ This was 1961 and presumably imported isotopes were not likely to be available for a local hospital in Tainan, and there was no domestically produced RAI yet. Perhaps Yan acquired it from Japan.^119^

By 1983, two physicians from Chang Gung Hospital, an enterprising private hospital established in 1976 during the height of surgical medicine, noted an increased use of RAI. The article provided details of the clinical use of RAI, including indications, patients’ age and sex, possible side effects, and the mechanism of RAI, implying that the use of RAI was not yet part of the establishment.^120^

From the point of view of internal and nuclear medicine, the use of RAI meant avoiding the risks posed by surgery. But as a radioactive substance, RAI implied a different kind of risk; both medical and lay people were concerned with the threat of cancer from radiation.^121^ Young children, pregnant women, and women who plan to become pregnant are not considered safe candidates for RAI treatment. Even though medical research in 1978 had stated that RAI did not increase the risk of cancer and it may even lower the risk of thyroid cancer, it was suspected of being cancer-causing.^122^ As late as 2003, the internist Tian-Chun Chang of National Taiwan University Hospital still lamented: ‘In Taiwan, whenever people hear the term radioactive iodine or atomic iodine, they retreat 100 miles [tuibi sanshe],^123^ and I am forced to tell them that it’s not an atomic bomb.’^124^

Even in the early twenty-first century, a well-respected endocrinologist working in a medical centre in southern Taiwan was still troubled by some rumours that had purportedly been made up by some surgeons working in local clinics. According to the rumours, RAI was ‘bad for the health’ due somehow to its atomic nature.^125^

## Conclusion

Eisenhower asserted in his Atoms for Peace speech that the ‘miraculous inventiveness of man shall not be dedicated to his death, but consecrated to his life’, and politicians, scientists, and physicians in Taiwan similarly commented on the marvels of the Atomic Age.^126^ As one of the ‘friendly nations’, Taiwan contributed to the picture of how life would be drastically improved.^127^ Needless to say, Taiwan was eager to develop nuclear science and the Atoms for Peace campaign was a forceful entry point. The use of iodine-131 to treat hyperthyroidism was part of this fantastical portrait of the future and the desire for nuclear modernity. The hyperbole germinated through this nuclear science imaginary and, given authoritative voice and urgency by Cold War political powers, created the expectation that iodine-131 would swiftly take over thyroidectomy. Yet, as we have seen, not every item in this international packaging of American hegemonic, anti-Communist nuclear science could be unpacked, fitted, and utilised locally.^128^

Along its ambitious global itinerary as a ‘miraculous’ phenomenon, RAI’s arrival and transformation as a knowledge and practice in Taiwan encountered several obstacles. The first was that postcolonial Taiwan was not a medical wilderness; colonial Taiwan had experienced medical modernity. Thyroid diseases such as goitre possessed significant scientific, symbolic, and practical value in colonial efforts at Japanese regional expansion. Japanese surgeons who studied goitre were also proficient in performing thyroidectomy, and after the war their legacy was carried on by their Taiwanese disciples. In postcolonial Taiwan, surgery became firmly established as one of the major forms of medical treatment for myriad diseases and conditions, including thyroid diseases. Surgical clinics were established wherever gaps existed in the local medical market. Even though nuclear science was critical for Taiwan’s state-building during the Cold War, non-state actors such as physicians and surgeons, as this paper shows, engaged in the multifaceted processes of the globalisation of isotopes.

In postcolonial Taiwan, Tian-Cheng Kao, the superintendent of NTUH, appeared to be enthusiastic in promoting isotopes, yet, in practice, most surgeons remained part of the colonial surgical tradition. Kao framed the new technology – isotopes – both as an import from the new dominant power and as a continuation of medical research and practice conducted under Japanese rule, which thereby asserted the professional legitimacy and expertise of the local medical and scientific elite. The geopolitics behind the promise of nuclear science, which made it into a Cold War science, had to contend with the legacy of Japanese colonial medicine and the practicalities of the medical market in postwar Taiwan. In the end, iodine-131 was an expensive and politically overwrought show pony in comparison with the facts on the ground of thyroidectomy.

The second obstacle to its failure to become a widespread practice in Taiwan has to do with the materiality of iodine-131 and the available infrastructure at the time. Before THOR could produce iodine-131, it had to be air-transported from the U.S., and its quick loss of efficacy presented real difficulties. It was also very expensive. It was not easily integrated into a clinical or professional niche. In fact, its use was limited to elite hospitals in Taipei such as NTUH and the Taipei Veterans General Hospital, and professional societies were slow in instituting it. Finally, in the popular imagination, iodine-131 was equated with the aftermath of the atomic bombing of Japan. There was indeed an analogy between atomic bombs and iodine-131, as in the 1957 ceremony in which the verb ‘dropped’ was used in the newspaper report.

Facing these obstacles, the U.S. and its allies’ best efforts to put a positive spin on going nuclear through a worldwide redemption tour – i.e., attempting to transform the horrors of the Atomic Bomb into the miracles of Atoms for Peace – failed. Encountering the local circumstances in Taiwan, iodine-131 hardly stood a chance as a real rival to surgery. This analysis of the history of iodine-131 in Taiwan shows how the globalisation of nuclear medicine has been uneven through time and space. As mentioned earlier in this paper, Korea and Taiwan share some similarities in the history of nuclear medical modernity. In both countries, currently antithyroid drugs are the first line of treatment for hyperthyroidism, as opposed to the U.S. where iodine-131 is preferred, but the two countries took different paths. For Korea, iodine-131 was the second line of treatment, but for Taiwan, it was surgery. It is not surprising that Korean physicians find it ‘difficult to apply’ management guidelines for hyperthyroidism produced by the American Thyroid Association and the American Association of Clinical Endocrinologists, as they are different from clinical practice in Korea.^129^ This may be in part due to goitre was not a serious issue in the past in Korea, as mentioned earlier in this paper.

The road to globalisation, whether smooth or uneven, was partly paved by the local circumstances. The imported notion of the useless and pathological uterus in Taiwan was a case in point – it was nurtured by the various programs such as family planning and cancer prevention campaign, and it went hand in hand with the practice of prophylactical hysterectomy.^130^ Iodine-131 faced more adverse circumstances. As knowledge moves, how does it survive or flourish, or fail to do so, in different localities? It depends on how much it can engage with the local conditions.

## Footnotes

2 ‘Fangshexian dian 131 yaowan zimei kongyun ditai zhibing [Radioactive Iodine 131, air-transported and arrived in Taiwan, to cure disease]’, *Lianhe Bao* [United Daily], 25 October 1957.

3 For a detailed guideline, see Douglas S. Ross , ‘Radioactive Therapy for Hyperthyroidism’, The New England Journal of Medicine, 364, 6 (2011), 542–550 10.1056/NEJMct100710121306240.

4 Yi-Lin Liang, ‘The Epidemiology of Hyperthyroidism in Taiwan’, MA Thesis, Institute of Clinical Medicine, National Cheng Kung University, 2013, 2.

5 Korea shared some similarities with Taiwan in the ways in which nuclear medicine began. Munho Lee , ‘The Present Status of Nuclear Medicine in Korea’, The Korean Journal of Nuclear Medicine, 2, 1 (1962), 9–14 .

6

Jae Hoon Moon and Ka Hee Yi , ‘The Diagnosis and Management of Hyperthyroidism in Korea: Consensus Report of the Korean Thyroid Association’, Endocrinol Metab, 28, 4 (2013), 275–910.3803/EnM.2013.28.4.275PMC387103624396691.

7

Shih-Ming Huang , Chen-Hsen Lee , Fang-Fu Chou , Koung-Yi Liaw , Tain-Chen Wu, and Taiwan Endocrine Surgeons Study Group, ‘Characteristics of Thyroidectomy in Taiwan’, J. Formosa Med. Assoc., 104, 1 (2005), 6–11 15660170.

8 Interview with Dr. Wen-Jen Yao, a practitioner in nuclear medicine at a medical centre in Taiwan. 8 September 2015.

9

John Krige , ‘The Peaceful Atom as Political Weapon: Euratom and American Foreign Policy in the Late 1950s’, Historical Studies in the Natural Sciences, 38, 1 (2008), 5–44 ; Angela N. H. Creager , Life Atomic: A History of Radioisotopes in Science and Medicine (Chicago: University of Chicago Press, 2013).

10
*Ibid.*, Creager, 137-8.

11

Mara Drogan , ‘The Atoms for Peace Program and the Third World’, Cahiers du Monde russe, 60, 2/3 (2019), 441–60.

12

John Krige , ‘Techno-Utopian Dreams, Techno-Political Realities: The Education of Desire for the Peaceful Atoms’, in Michael D. Gordin , Hellen Tilly , and Gyan Prakash (ed.), Utopia Dystopia: Conditions of Historical Possibility (Princeton, NJ: Princeton University Press, 2010), 151–75.

13

Gisela Mateos and Edna Suárez-Díaz , ‘Technical Assistance in Movement: Nuclear knowledge Crosses Latin American Borders’, in John Krige (ed.), How Knowledge Moves: Writing the Transnational History of Science and Technology (Chicago: University of Chicago Press, 2019), 345–67.

14 Saul Hertz (1905–1950) and A. Roberts pioneered iodine-131 in 1942. A. J. Lowery and M. J. Kerin , ‘Graves’ Ophthalmopathy: The Case for Thyroid Surgery’, Surgeon, 7, 5 (2009), 290–610.1016/s1479-666x(09)80007-319848063; Krige, *op. cit.* (note 12).

15 Hyperthyroidism, also known as Graves’ disease, is ‘caused by autoantibodies to the thyroid gland that mimic thyroid-stimulating hormone, causing the gland to overproduce thyroid hormone’. Robert Gensure (ed.), *Graves’ Disease* (London: United Kingdom, 2021). Goiter is defined as the ‘enlargement of the thyroid gland that may be associated with changed function of the thyroid that can result from under-production or over-production of hormone or from a deficiency of iodine in the diet’. ‘Goitre’ in W. Kirch (ed.), *Encyclopaedia of Public Health* (Dordrecht: Springer). https://doi.org/10.1007/978-1-4020-5614-71287, accessed 14 June 2024.

16

Yu-Ling Huang , ‘Biopolitical Knowledge in the Making: Population Politics and Fertility Studies in Warly Cold War Taiwan’, East Asian Science, Technology and Society: An International Journal, 10, 4 (2016), 377–99.

17

Honghong Tinn , Island Tinkerers: Innovation and Transformation in the Making of Taiwan’s Computing Industry (Boston: The MIT Press, 2025).

18

Wayne Soon , Global Medicine in China: A Diasporic History (Stanford: Stanford University Press, 2020).

19

Harry Yi-Jui Wu , Mad by the Millions: Mental Disorders and the Early Years of the World Health Organization (Boston: The MIT Press, 2021).

20 Shinyi Hsieh, Pluralizing Postcoloniality from a Standpoint of Margins: A Historical Detour of Global Health and Imperial Regimes in Postwar Taiwan through the Traces of the Marginalized (PhD Dissertation in History of Health Sciences, UC San Francisco 2023); Shinyi Hsieh , ‘Navigating Geomedical Borders NAMRU-2’s Tropical Operation in Taiwan and Southeast Asia, 1955-1979,’ Taiwan: A Radical Quarterly in Social Studies 131 (2024), 77 . An earlier work on postcoloniality is Kuan-Hsing Chen, *Asia as Method: Toward De-imperilization* (Durham, NC: Duke University Press, 2010).

21 The National Defence Centre relocated to Taipei from Shanghai in 1949. In the period between 1954 and 1962, four private medical colleges were established whose faculty members came mostly from the Medical College of National Taiwan University, formerly Taipei Imperial University. Tung-Liang Chiang , ‘Physician Manpower Policy in Taiwan, 1945-1995’, Chinese Journal of Public Health, 14, 5 (1995), 383–91.

22

Tsugio Horiuchi , ‘Honjima no chihōbyō ni kan suru ni san no umfurage teishutsu oyobi setsumei [Endemic Diseases of this Island—Presenting and Explaining]’, Journal of the Formosan Medical Association, 1, 5 (1902), 42–4.

23 Tomoe Takaki, *Die hygienischen verhaltnisse der insel Formosa* (Dresden, 1911) Published for the International hygiene exhibition.

24

Yoshio Hashimoto , Chinio Kobayashi , Eiraku Ri [Yong-Le Li], Yoriaki Sasaki , Ekkyo Sen [Yi-Gong Zhan], ‘The Preliminary Report on the Prophylaxis against Endemic Goiter: On the Iodine Prevention of School Children in Taiwan, Japan’, JFMA, 41, 451(1942), 106–11.

25 After the war, Kunio Kawaishi returned to Hiroshima, and he also began to use isotopes to study thyroid and treat hyperthyroidism. Kaori Iida , ‘Peaceful Atoms in Japan: Radioisotopes as Shared Technical and Socio-political Resources for the Atomic Bomb Casualty Commission and the Japanese Scientific Community in the 1950s’, Studies in History and Philosophy of Science, 80 (2020), 1–10 10.1016/j.shpsc.2019.10124031883712.

26

Yoshio Hashimoto , ‘Kawaishi Sensei and I’, in Memorial Committee for Kunio Kawaishi (ed.), *Kunio Kawaishi* (Hiroshima: Second Surgical Division, Faculty of Medicine, Hiroshima University, 1975), 156 .

27 For instance, Magoichi Itaya, the superintendent of Itaya Hospital in Taipei, was granted a doctoral degree for his research on the pathology of endemic goitre by Kyoto University. ‘Taiwan yixuejie de mingyu [Honor in the Medical Community in Taiwan]’, *Taiwan gongzhong yishi zazhi*, 4, 7 (1931), 27; Guo-Ming Tsai, the founder of Tsai Waike (Tsai’s Surgical Clinic established in 1949) in Kaohsiung, also received a degree from Kyoto University for his research on goitre under Kawaishi. Kaohsiung City Medical Association (ed.), *Kaohsiung Yiliao Shih* (Medical History in Kaohsiung) (Kaohsiung, 1998), 137; With his seven papers on goitre, Ikung Chan, a practitioner in Shinchiku Prefecture in colonial Taiwan, received his PhD also under Kawaishi in 1945. ‘Chan Ikung Shi shuo shuo yi bo [Ikung Chan Receives PhD in Medicine]’, *Ming Pao*, 6 December 1945.

28

Yen-chiou Fan , ‘Imperial Politics and Medicine: The Medical Faculty in Taihoku Imperial University under Japan’s Wartime Total Mobilization’, Bulletin of Taiwan Historical Research, NTNU, 1 (2007), 89–136 .

29 Hashimoto et al., *op. cit.* (note 26), 106.

30

Kunio Kawaishi , ‘Taiwan ni o ke ru chihousei kojo senshu [Endemic Goiter in Taiwan]’, Journal of Formosan Medical Association, 39, 10 (1940), 111 . However, in his commemorative essay on Kawaishi, Ikung Chan, one of Kawaishi’s Taiwanese disciples, writes about how he was struck by Kawaishi’s lecture that began with the well-known artist Tenshin Okakura’s notion of ‘Asia as One’ but was ambivalent about the war. It seems in the context of classroom, as a teacher Kawaishi’s attitude towards Japanese expansion was not as strong as in writing, and he even recognised the war as a mistake. Ikung Chan, ‘Ajia wa hitotsu nari [Asia is one]’, in Memorial Committee for Kunio Kawaishi (ed.), *Kunio Kawaishi* (Hiroshima: Second Surgical Division, School of Medicine, Hiroshima University School, 1975), 178.

31 Hashimoto and his colleagues write: ‘Once we turn our attention to the [greater] East Asian Co-Prosperity Sphere and receive reports of the spread of the disease [goitre] in the southern islands, our nation’s southern policy should first observe in detail the situation of the disease in Taiwan, which is close to it, and use this as a basis to establish its prevention methods. This is extremely important right now’. Hashimoto et al., *op. cit.* (note 24), 106.

32

Kenji Fujibayashi , ‘Kōjōsen shu no chihōbyōgakuteki oyobi jikken-teki jikō [Endemic and Experimental Aspects of Goitre]’, Journal of Formosan Medical Association, 13 (1914), 18–60 . Kenji Fujibayashi , ‘Hualien Poto no Kōjōsen-shu Haifu jōkyō [The Distribution of Goitre in Hualien Port Area,’ Journal of Formosan Medical Association, 12 (1913), 26–47 . Goshichirō Matsuyama , ‘Jukirin no kōjōsen kanja [Goitre patients in Shujilin]’, Journal of Formosan Medical Association, 3 (1904), 42–4. Fujibayashi (1879–) served as the superintendent at various colonial hospitals throughout his career in Taiwan, including Taiwan Governor General Hospital, Taipei Hospital, Tainan Hospital, Hualien Port Hospital, and Takao Hospital. He took on private practice after he retired in 1919.

33 World Health Organisation, *Endemic Goitre*, World Health Organization Monograph Series, No. 44, p. 182.

34

Shigenori Takenaka , ‘Nihon no Kōjōsen-shu [Goitre in Japan],’ Journal of Formosan Medical Association, 13, 144 (1914), 1086–8. In this article, Takenaka referred to his article published in *Chugai Ijishimpo* in 1899. However, he also noticed that the ‘patients’ did not see goitre as a disease.

35

Kunio Kawaishi and Yoshio Hashimoto , ‘Endemic Goitre in Japan’, Annals of Surgery 113, 4 (1941), 481–9510.1097/00000658-194104000-00001PMC138637317857750.

36 For example, Nakao Ito’s pathological studies on 42 goitre patients in Jehol. Nakao Ito , ‘Endemic Goitre in Jehol: Report II. The Non-protein N in the Blood’, Folia Endocrinologica Japonica, 11, 9 (1935), 971–86.

37

World Health Organization, Endemic Goitre (Geneva: World Health Organization, 1960), 177–8.

38 Kubo’s research subjects were those who belonged to anti-Japanese organizations (called ‘bandit’ by the Japanese) from Rehe (or Jehol) and had been sentenced to death. He examined them for the presence of goitre and interviewed them for clues of possible causes. Kubo dissected the corpses and took pieces of the thyroid from each of them as specimens. Keiko Suenaga , ‘Bodies in the Service of the Japanese Empire: Colonial Medicine in Manchuria’, The Asia-Pacific Journal, 19, 24, 3 (2021), 1–22 .

39 Tien-Jun Chang, *Dangdai Yixue* [Contemporary Medicine], 33, 12, (2006), 953–5; Xi-Yu Fang, ‘Yi Kawaishi Kunio Jiao-shou [Remembering Professor Kunio Kawaishi’, in *Taida yiyuan bainian huaijiu* [One Hundred Years of National Taiwan University Hospital], (Taipei: National Taiwan University Hospital, 1995), 25. Fang was one of Kawaishi’s disciples and a prominent surgeon flourished in the post war era.

40 Tian-Yu Lin, *Xinlin Shengya Yun ho Yueh* [My Career in Medicine] (Taipei: Commercial Press, 1990).

41 For example, Dr. Guo-Ming Tsai who established the Tsai Surgical Clinics in Kaohsiung in 1949 studied goitre under Kawaishi. Kaohsiung Yishi Gonghui [Kaohsiung Medical Association] (ed.), *Kaohsiung Yiliao Shih* [Medical History in Kaohsiung] (Kaohsiung: Kaohsiung Yishi Gonghui), 138. Tian-You Lin (1913–1995), a surgeon at NTU Hospital for over four decades, was a disciple of Sawada. Tian-Yu Lin, *Xiangya zhita chunqiuji* [Lin’s autobiography] (Taipei: Commercial Press, 1993). Bang-Xing Xu, whose Di-Yi Waike [First Surgical Hospital] was very prosperous, subsequently opened Xu Waike in Kaohsiung. He was also a disciple of Sawada.

42 Tien-Cheng Kao was not educated in Taihoku Imperial University and did not study under Kawaishi and Sawada, but his education in Tokyo Imperial University created in him a sense of affinity with the Japanese past. After assuming the position of the superintendent of the NTU Hospital in 1953, he became an important leader in the medical community in Taiwan.

43 There is no comprehensive nationwide survey available on the establishment of surgical clinics. However, a look at the city of Tainan gives a sense of the historical development of surgical practice – except for Tainan Hospital (established in 1896) and Sin-lâu Hospital (established in 1869 by the Presbyterian missionary James Maxwell), private surgical clinics began to burgeon in the mid-1950s. Hsiu-Yun Wang (ed.), *Discovering Medical Tainan* (Tainan: STM Centre, 2022).

44 Tu’s congratulatory message, in National Taiwan University Hospital, Kao Tien-Cheng jiaoshou renzhi shizhounian jinian ce [Professor Tien-cheng Kao’s Tenth Anniversary Commemorative Album] (Taipei: National Taiwan University Hospital, 1959).

45 ‘List of Colleagues of Surgical Department’, in National Taiwan University Hospital, *Kao Tien-Cheng jiaoshou renzhi shizhounian jinian ce* [Professor Tien-cheng Kao’s tenth anniversary commemorative album], Taipei: National Taiwan University Hospital, 1959.

46 According to Kawaishi’s survey published in 1940, its prevalence was 6.68% in Taipei, Taiwan, compared to 0.88% in Sapporo, Japan. The same survey also compared different ethnic groups in colonial Japan – Japanese 18%, Taiwanese (*bendao ren*, ethnic Chinese who immigrated to Taiwan before 1895) 44.70%, and Aborigines (Austronesian) 61.10%. Kunio Kawaishi , ‘Taiwan ni o ke ru chihousei kojo senshu [Endemic Goiter in Taiwan]’, Journal of Formosan Medical Association, 39, 10 (1940), 111–72. Taiwan initiated an iodine-salt project in 1958 and full coverage was completed in 1967, after which the goitre problem greatly improved. Yet, it did not completely vanish, and public health experts pointed to the problem of the source of drinking water. Island-wide running water did not become available until the late 1980s. Tien-Jun Chang , ‘Retrospective and Prospective of Endemic Goitre in Taiwan’, Journal of Internal Medicine of Taiwan, 11, 2 (2000), 51–56.

47

Tsung-Po Kuo , ‘Treatment of Thyroid Crisis’, JFMA, 55, 8–9 (1956), 23–45 .

48 Kaohsiung City Medical Association (ed.), *Kaohsiung Yiliao Shih* (Medical History in Kaohsiung) (Kaohsiung, 1998), 142.

49 Mentions of thyroid disorders can be found in the diaries of Xin-Rong Wu (14 January 1957), Xian-Tang Lin (19 May 1943), Wang-Cheng Huang (12 November 1927), Lijun Zhang (4 September 1909). Taiwan riji zhishiku [Taiwan Diary Database], https://taco.ith.sinica.edu.tw/tdk/, accessed 15 May 2024.

50 ‘Senior Doctor of Adventist Hospital flew to Lybia’, *Lianhe Bao*, 18 June 1957.

51 Harry Miller Collection, Centre for Adventist Research, James White Library, Andrews University, Michigan, 2008.

52 Yu-Ching Sheng was probably one of the first few who wrote a detailed account of her illness as a hyperthyroidism patient in 1935. Yu-Ching Sheng, ‘Gengsheng Ji [Coming Back to Life]’ in Yu-Ning Li (ed.), *Jidai Chunghua zixu shiwen xuan* [Anthology of women’s autobiographical accounts in Modern China] (Taipei: United Publishing, 1980), 715-41. Daizu Ye , ‘huigu liushi nian qian zai baerdimode shinian [Remembering my ten years in Baltimore 60 Years ago]’, Yuanyuan Jikan, 46 (2013), 54 .

53

Hsiu-Yung Wang , ‘The Making of the “Useless and Pathological” Uterus in Taiwan, 1960s-1990s’, Medical History, 65 (2021), 46–69 .

54

Zu-Miao Zhao , Choulou de Yisheng [Ugly Doctors] (Taipei: Siji Publishing, 1978), 53 , 54.

55 ‘Certain doctors use the knife at will, patients with Labourer’s insurance let them cut women labourers’ thyroid, legislative member urged the government to take action’, *Zhongguo Shihbao*, 8 November 1977.

56 Tai-an Zheng, M.D., ‘Thyrotoxicosis’, *Zhongguo Shihbao*, 17 December 1978.

57 ‘Spare the knife, thyroid tumours may be malignant or benign, not all require surgery’, *Zhongguo Shibao*, 5 March 1980.

58 Feng Li, ‘Bubiyao de kaidao women xuyao lifa zhicai,’ [For unnecessary surgeries we need legislative sanctions] in her *Shimao shehui de shimao bing* [The fashionable diseases of fashionable society] (Taipei: TTV Cultural Enterprise Ltd, 1982), 45–49.

59 Wang, *op. cit.* (note 53).

60 Shu-Chuan Li, ‘Jiazhuangxian kangjin zhiliao baipishu [White book for treatment of hyperthyroidism]’, *Mingsheng Ribao*, 26 March 1994.

61 Wang, *op. cit.* (note 53).

62 In 1949, Kuomingtang retreated to Taiwan after losing the civil war to Communists and became part of the ‘Free’ world.

63 Chi-Yun Chang, ‘kexue yu zhengzhi: minguo 45 nian 12 yue 25 ri huanying wanguo gwaike xuehui huiyuan zhici [Science and Politics: Welcome Remarks at International Surgical Society, 25 December 1956’, in Chang Chiyun Xiansheng Wenji [Anthology of Chi-Yun Chang’s Writings] vol.18 (Taipei: Chinese Culture University), 9475.

64

Chi-Yun Chang , ‘Atomic Energy and the Reconstruction of China’, in The Essence of Chinese Culture (Taipei: China Academy, 1957), 358 . A welcoming address at a reception in honour of the five scientists of the American Atomic Energy Commission, 15 June 1957.

65

John Krige , ‘Atoms for Peace, Scientific Internationalism, and Scientific Intelligence’, OSIRIS, 21 (2006), 178 .

66 Agreement Between the International Atomic Energy, The Government of Republic of China and the Government of the United States of America for the Application of Safeguards (6 December 1971). URL: https://law.moj.gov.tw/ENG/LawClass/LawAll.aspx?pcode=Y0070290. United States Atomic Energy Commission, *Nineteenth Semi-annual Report of the Atomic Energy Commission*, January 1956, p. 132. Appendix 4 ‘Shipments of Radioactive and Stable Isotopes to Foreign Countries’.

67 Those who received American aid to go abroad included Tien-Cheng Kao, Kuang-Chu Wang, Shu Yeh (pathologist). Marshall Brucer mentioned in the preface of one of his ORIN reports that Wang run some of the experiments for thyroid radioiodine uptake measurement. Marshall Brucer, ‘Thyroid Radioiodine Uptake Measurement: A Standard System for Universal Intercalibration’, Oak Ridge Institute of Nuclear Studies, Oak Ridge, Tennessee, 1959.

68 ‘Radioactive substances for Medical Purposes, Taidai Hospital Research Centre Breaks Ground Today’, *Lianhe Bao*, 12 June 1957.

69 A merge of the former Beiyang Military Medical School (established in 1902) and war-time medical training program in China formed National Defense Medical Center in 1947. Wayne Soon , Global Medicine in China: A Diasporic History (Stanford: Stanford University Press, 2020). For the early history of NDMC and role of Robert K.S. Lim, see Shi-yung Liu , Shih-ching Kuo , ‘Robert K.S. Lim (1897-1969): Academician of Academia Sinica and Chancellor of National Defence Medical Centre in Silent Shadow’, Taiwan Historical Research, 19, 4 (2012), 141–205 .

70 Chi-Yun Chang, ‘Xingzheng yuan yuanzineng weiyuanhui liangnian lai zhi gongzuo [The work of the Atomic Council of Executive Yuan for the Past Two Years]’, in *Chang Chiyun Xiansheng Wenji* [Anthology of Chiyun Chang’s Writings] vol. 18 (Taipei: Chinese Culture University), 9484-8.

71

Shinyi Hsieh , ‘Navigating Geomedical Borders NAMRU-2’s Tropical Operation in Taiwan and Southeast Asia, 1955-1979’, Taiwan: A Radical Quarterly in Social Studies, 131 (2024), 77 .

72 United States International Cooperation Administration, Office of Public Health, *ICA Health Summary* (Washington, 1958), 10.

73 Among those who were trained at NDMC, including Yu-Zhen Guan (1922–), Jun Yu (1925) were all specialized in X-ray. Guan was a graduate of Xiang-Ya Medical College. Under the influence of Jing Wu, she switched from internal medicine to radiology, as she thought ‘there would be no hospitalized patients’. ‘Interview with Yu-Zhen Guan’, 69-107 in *Fifty Years at Taipei Veterans General Hospital: Oral Histories. Part II; Department Chairs and Professors* (Taipei: Institute of Modern History, Academia Sinica, 2011)

74

Tien-Cheng Kao . ‘Dr. Jing Wu—A Pioneer in Atomic Medicine in Free China’, Zhongguo Yizhou, 349 (1956), 7.

75 Jing Wu interned and did residency at Peking Union Medical College for 4 years (1926–1930). Jing Wu, ‘Wo yu fangshexian yixue liushinian [I and Radiology for Sixty Years]’, (author’s own print, 1988).

76 For example, in a correspondence between Chih-Teh Loo and Dr. O. R. McCoy (CMB) in 1965, Loo requested a radio-isotope counting equipment for teaching purposes. Chih-The Loo to Dr. O. R. McCoy, November 22 1965, Rockefeller Archive Center, China Medical Board Records, RG2, Accession 2014:022, SG1 (FA1292), Box 69, Folder 1106, ‘National Defense Medical Centre (NDMC), General Correspondence, Director, and Staff’. I thank Hsiao-Ling Chen for sharing this file.

77 During this time Wu worked in various places – during 1955–1956 he was at Albert Soiland Cancer Foundation and in 1956 he was at Newton Wellesley Hospital. Though he claimed that he studied atomic energy in medicine, his publications revealed that he remained in radiology. Wu, *op. cit.* (note 75), 3.

78 Chung-Chuan Zhang, *Wuyi wen changtien: Wo de huiyi lu, 1925-2011* [No intention to ask the heaven – My Memoir] (Taipei: Shu-Chuan, 2012), 142.

79 ‘Taidai yiyuan jian yuanzi yixue yanjiushi xing potuli, [Groundbreaking Ceremony for the atomic medicine lab of NTU Hospital]’, *Kexue Huibao*, 7, 1 (1957), 21. Marshall Brucer was the guest of honour at the event. United States International Cooperation Administration, Office of Public Health, *ICA Health Summary* (Washington, 1958), 14.

80 Tien-Cheng Kao et al, ‘I-131 Thyroid uptake study of Endemic Goiter in Taiwan, China’, Proceedings of the second United Nations International Conference on the Peaceful Uses of Atomic Energy, held in Geneva, 1 September–13 September 1958, Vol. 26, Isotopes in Medicine, 73.

81

Tien-Cheng Kao , Kuang-Chu Wang , and Hsueh-Fang Yang , ‘Iodine-131 Thyroid Uptake Study of Endemic Goiter Patients in Taiwan’, International Journal of Applied Radiation and Isotopes, 7 (1960), 299–305 10.1016/0020-708x(60)90118-614404348.

82

Tien-Cheng Kao , ‘Clinical Use of Radio-isotopes in Surgery’, JFMA, 57(1958), 55–79 . To be sure, Kao was not the first one to publish on isotopes in JFMA. In 1948, Yuji Hosoya published ‘The use of isotope in Medicine’ and Kuan-Chu Wang published ‘The problem of the use of radioactive isotopes’ in 1957.

83 At National Taiwan University Hospital, the radiotherapy/teletherapy machines such as cobalt-60, depending on the situation, were run by both radiologists and nuclear radiologists, though there was tension among the two groups for at least until the late twentieth century.

84 Other achievements were a nursery, residents’ dormitory, and the completion of the repair of the Dietary Department. National Taiwan University Hospital (ed.), Guoli taiwan daxue fushe iyuan yuanzheng Kao Tien-chang jiaoshou renzhi shizhounian jinian ce [Ten Year Anniversary for Professor Tien-Cheng Kao, Superintendent of National Taiwan University Hospital] (Taipei: National Taiwan University Hospital, 1959).

85

Tien-Cheng Kao , ‘Wu Jin boshi: ziyou zhongguo yuanzi yixue de tuohuangzhe [Dr. Jin Wu: The pioneer in atomic medicine in Free China]’, Zhongguo Yizhou, 349 (1956), 7–8 ; Tien-Cheng Kao , ‘Aizheng huanzhe de xingjiu xing [Cancer Patients’ new savior]’, Zhongguo Yizhou, 416 (1958), 4–5 ; Tien-Cheng Kao , ‘Fangshexing tongweisu zi zhenliao yongtu [the use of radioactive isotopes in diagnosis and treatment]’, Dianzi kexue yu jishu faming zhanlan zhuanhao [Special issue for exhibition on the innovations in electronic science and technology], Jiaoyu yu wenhua [Education and Culture], 174 (1958), 2–17 .

86 National Taiwan University Hospital, Kao Tien-Cheng jiaoshou renzhi shizhounian jinian ce [Professor Tien-cheng Kao’s Tenth Anniversary Commemorative Album] (Taipei: National Taiwan University Hospital, 1959), 13.

87 ‘Taiwan sheng yixuehui di shisi jie nianhui [The 14th Meeting of the Medical Association of Taiwan Province]’, *Lianhe Bao*, 23 November 1959.

88 ‘Atomic Medicine for Heart Diseases’, *Lianhe Bao*, 17 July 1960.

89 ‘The Development of Radiology in Today’s World’, *Lianhe Bao*, 11 April 1953.

90

Yang Xue-Fang , ‘Hyperthyroidism: Using Anti-thyroid Medication as a Treatment in Internal Medicine’, Journal of Formosa Medical Association, 55, 7-8 (1956), 17 . A similar attitude can be observed in the article co-authored by two surgeons. As they did not have the experience of using iodine-131, they merely introduced the topic as a new development. See, Tsung-Po Kuo and Sha-Nan Huang , ‘Gezhong linchuang jianyan dui jiazhuangxian jibing zhenduan zhi jiazhi [The value of various clinical tests for the diagnosis of thyroid diseases]’, JFMA, 55, 8-9 (1956), 46–67 .

91

Hsueh-Fang Yang , Kuang-Chu Wang , Yao Chang-Chien , Jui-San Chen , Chao-Ming Ho , and Tien-Cheng Kao , ‘An Evaluation of I-131 Treatment of Hyperthyroidism: Analysis of 114 Cases’, JFMA, 59, 10 (1960), 142 .

92 Chuan-Ching Chen, ‘Yuanzineng zai yiliao shang de yingyong [The medical applications of atomic energy]’, Yuanzineng weiyuanhui huibao [Atomic Energy Commission bulletin], 1, 2 (1965), 17-27.

93
*Jingji Ribao*, 4 March 1970.

94 Dr. Wen-Long Chen, an ob-gyn, in his popular essay on hyperthyroidism also supported the use of iodine-131. Wenlong Chen, ‘Jiyu chengji: Jiantan jiazhuangxian jineng kangjinzheng [Becoming sick from accumulating depression: On Hyperthyroidism]’, *Zhongyang Ribao*, 2 December 1966.

95 Kao Tien-Cheng, ‘Taida Yiyuan Yuanzhang Kao Tien-Cheng jiaoshou xieci’ [Acknowledgments from Professor Kao Tien-cheng, Superintendent of National Taiwan University Hospital], in National Taiwan University Hospital, Kao Tien-Cheng jiaoshou renzhi shizhounian jinian ce [Professor Tien-cheng Kao’s Tenth Anniversary Commemorative Album] (Taipei: National Taiwan University Hospital, 1959), 13.

96 For a history of colonial medicine in Taiwan, see Michael Shiyung Liu , Prescribing Colonization: The Role of Medical Practices and Policies in Japan-Ruled Taiwan, 1895-1945 (Ann Arbor: Association for Asian Studies, 2009).

97

James Cameron , M. Cohen , E. Eriksson , Richard Olsen , B.R. Payne , Henry Seligman , and Herbert Vetter , ‘The Use of Radioisotopes in Developing Countries’, International Atomic Energy Agency Bulletin, 5, 2 (1963), 25–28 .

98 ‘Taipei Business Information’, *Zhengyang Ribao*, 27 July 1957.

99 Indirectly related to the cost of the Iodine-131 treatment, in 1957, with NTUH’s own funds, U.S. aid and Tsinghua University’s special funds, Kuan-Chu Wang purchased a ‘complete set of thyroid measuring instruments’ that cost 7,000 USD. History of the Department of Nuclear Medicine at National Taiwan University Hospital, https://www.ntuh.gov.tw/nm/Fpage.action?muid=5913&fid=1312, accessed 22 September 2024.

100 Although there are no records for the cost of thyroidectomy, local medical professionals regularly issued fee standards. For operations, there were only three categories – small, intermediate, and major. According to fee standard issued by Tainan Medical Association in 1949, a small operation was 20 NT, intermediate operation was 150 NT, and major operation was 300 NT. A thyroidectomy was probably in the category of intermediate surgery, or between intermediate and major, depending on the situation. Tainan Medical Association, ‘Medical Expense Regulations’, 1949. Kaohsiung Medical Association, ‘Medical and Pharmaceutical Cost Schedule’, 1949.

101 By 1982, thyroidectomy was 900 NT for contracted hospitals but was 6500 NT for major hospitals such as National Taiwan University Hospital and the Veterans General Hospital, a gap that caused a controversy. *Lifa Yuan Gongbao* [Legislative Yuan Gazette], 76, 44 (1987), 112. In the 1990 version, there were unilateral subtotal thyroidectomy (4070 NT), bilateral subtotal thyroidectomy (7200 NT), excision of thyroid cyst or thyroglossal cyst (4040), and total thyroidectomy (5430 NT). Ministry of Interior, *Taimin diqu Laogongbaoxian zhenliao feiyong zhifu biaozhun biao* [Diagnosis and Treatment Payment Standards for the Labor Insurance in Taiwan and Min-nan Areas] (1960, 1977). Council of Labour Affair, Executive Yuan, *Laogongbaoxian zhenliao feiyong zhifu biaozhun biao* (1990), 156.

102
*Lienhe Bao*, 25 October 1957.

103 ‘Fangshexing dian 131 yaowan zi mei kongyun ditai zhibing, liu jiazhuangxian huanzhe shou huo zhiliao, Zhang Chi-Yun zuo qinjiang yaowan wei bingren fuyong [Radioactive iodine 131 capsules arrived in Taiwan by air from the United States for treatment. Six patients with thyroid disease were treated for the first time. Chi-Yun Chang personally delivered the capsules to the patients yesterday.]’, *Lianhe Bao*, 25 October 1957.

104 Chien-Chang Lin, ‘Tsing Hua yuanzilu sishinian qian de huiyi [Reminiscence on the Tsing Hua nuclear reactor 40 Years ago]’ in Zihua Li (ed.), *Guoli Tsing Hua daxue shuichishi fanyingqi yunzhuan sishinian huigu jinian zhuanji* [40 Anniversary of Operation of the National Tsing Hua University Nuclear Reactor] (Hsinchu: National Tsing Hua University, 2001), 18.

105 Wet method, https://inis.iaea.org/search/search.aspx?orig_q=RN:38022907, accessed 15 May 2024.

106 Junmíng Huang (ed.), *Yuanzi Neng Yu Tsinghua* [Atomic Energy and Tsinghua University] (Hsin-Chu: National Tsing-Hua University Press, 2011), 286-8.

107

Jui-Yun Mu , ‘Radioimmunoassay Studies for Thyroid Stimulating Hormone (TSH) in Rat Serum’, The Chinese Journal of Physiology, 21, 1 (1971), 37–44 . The radioactive iodine used in this study was from Dainabot Radioisotope Lab in Tokyo, Japan.

108 Institute of Modern History (ed.), *Fifty Years at Taipei Veterans General Hospital: Oral Histories Part III* (Taipei: Institute of Modern History, Academia Sinica, 2011), 96-7. In comparison, by 1962 there were 11 hospitals and medical institutes that had radio-isotope facilities in Korea. Lee, *op.cit.* (note 5).

109 Zhongchuan Chang. *Wuyi wen cangtian: Wode huiyilu, 1925-2011* [My memoir, 1925-2011] (Taipei: Shuchuan, 2012). Chang, an ob-gyn at Chung-Xing Hospital, received the Cobalt 60 machine after the Yuanziyixue shiyanyuan closed down in 1957.

110 During the 1960s the question of whether nuclear medicine was a discipline in its own right was being debated. See Herbert Vetter , ‘Nuclear Medicine—A New Discipline’, IAEA Bulletin, 8–2, (1966), 7–13 .

111 Interview with Wei-Jen Yao, *op. cit.* (note 8).

112

Xiao-Yi Zhao , ‘Shangdao fangshexian yixuejia Wu Jing boshi [Remembering Medical Radiologist Dr. Jing Wu]’, Zhuanji Wenxue, 59, 6 (1991), 79 .

113

Xing-Hua Ye , ‘Hezi yixue xiankuang yu zhanwang [Nuclear medicine: Current status and its future]’, Dangdai Yixue, 2, 5 (1975), 13–22 . Ye established the Nuclear Medicine Centre at the Veterans’ Hospital in 1971. Also, Kai-Yuan Tzen , ‘Nuclear Medicine—Yesterday, Today, and Tomorrow’, Chinese Journal of Radiology, 4 (1979), 237–42.

114 I have found only one mention of the use of a certain ‘set of thyroid uptake measurement equipment.’ See the history of the department of Nuclear Medicine, National Taiwan University Hospital, https://www.ntuh.gov.tw/nm/Fpage.action?muid=5913&fid=1312

115 Xing-Hua Ye, *op. cit.* (note 113). Louis Tak Lui , Lai-Lei Ting , Po-Ming Wang , Lily Fong , and Na-Na Chung , ‘Development of Radiation Therapy at National Taiwan University Hospital’, Therapeut. Radiol. Oncol. 2 (1995), 157–60.

116 Michael Adas has shown how technology, particularly machines, emerged as the measure of achievement of ‘man’ since the nineteenth century. In the case of nursing history, Sandelowski has also demonstrated how technology shaped nursing and nursing practice. Michael Adas , Machines as the Measure of Men: Science, Technology, and Ideologies of Western Dominance (Ithaca, New York: Cornell University Press, 1990); Margarete Sandelowski , Devices and Desires: Gender, Technology, and American Nursing (Chapel Hill: University of North Carolina Press, 2000).

117

Kuang-Yao Chen , ‘History of Diagnostic and Therapeutic Radiology in Mainland China and Taiwan’, Therapeut. Radiol. Oncol., 2 (1995), 1–10 . For a classical analysis of artifacts, see Wiebe E. Bijker , Of Bicycles, Bakelites, and Bulbs: Toward a Theory of Sociotechnical Change (Boston: The MIT Press, 1997).

118 Advertisement Section, *Taiwan Xingwen Bao*, 5 November 1961.

119 Abbott, a company that produced nuclear pharmaceuticals, began to ship radioactive iodine to its customers in 1952. Dr. Yan could have purchased from Abbott, but it’s uncertain. *A Promise for Life: The Story of Abbott* (2013) https://webstorage.abbott.com/PromiseForLife/EN/index.html, accessed 19 March 2024.

120 Kai-Yuan Tseng, Miao-Chu Huang, ‘Fangshexing dian 131: Zhiliao jiazhuangxian jinengkangjin zheng [Radioactive Iodine-131: Treatment for Hyperthyroidism]’, *Dangdai Yixue*, 10, 6 (1983), 521-2.

121 Interview with Yao, *op. cit.* (note 8).

122

William H. Beierwaites , ‘The Treatment of Hyperthyroidism with Iodine-131’, Seminars in Nuclear Medicine, 8, 1 (1978), 95–103 10.1016/s0001-2998(78)80010-576339.

123 In ancient time a She is equal to 30 li, and 1 li is about 1800 metres. Therefore, three Shes would be about 100.6 miles.

124

Tian-Jun Chang , ‘Yong fangshexing dian zhiliao jiazhuangxian jineng kangjin [using radioactive iodine to treat hyperthyroidism]’, Dangdai Yixue [Contemporary Medicine], 30, 11 (2003), 873–4.

125 Personal communication with an endocrinologist who has been in practice for over three decades in a medical centre in Kaohsiung city, June, 2024.

126 Dwight D. Eisenhower, Address Before the General Assembly of the United Nations on Peaceful Uses of Atomic Energy, New York City. 08 December, 1953. https://www.presidency.ucsb.edu/documents/address-before-the-general-assembly-the-united-nations-peaceful-uses-atomic-energy-new. Accessed date: 7 June 2022.

127 U.S. Department of State, ‘Peaceful uses of Atomic Energy’, Department of State Publication, 5888, Released June 1955.

128 In the case of using isotopes as a treatment for cancer, the trajectory was somewhat different. As Daiwie Fu has pointed out that, before 1968, NTUH primarily used radiation to treat cervical cancer, in contrast to the radical hysterectomy performed by Chien-Tien Hsu (1913–1992) of Taipei Medical University. His analysis of the social conditions necessary for the development of surgical approach is illuminating, but he does not explain why NTU preferred radiation. As the example of iodine-131 and hyperthyroidism in this article demonstrates, atomic energy for peace and Cold War politics may serve as a key driver of NTUH’s active development of isotopes as a treatment method, and when American aid terminated in 1966, the surgical approach found its way back. Daiwie Fu, *Yaxiya de Xinshenti* [Assembling the New Body: Gender/Sexuality, Medicine, and Modern Taiwan] (Taipei: Social Publishing, 2005)

129 Jae Hoon Moon and Ka Hee Yi, *op. cit.* (note 6), 275.

130 Wang, *op. cit.* (note 53).

## Long descriptions

### Figure 1. Long description

On the left, a map of Taiwan displays orange dots representing surgical clinic locations, with the highest concentration in the northern region near Taipei. A dashed line connects to a magnified inset of Taipei City on the right. In the Taipei inset, red dots mark individual clinic sites, densely clustered in central and western districts, with some spread into adjacent areas. The inset clarifies the urban concentration compared to the broader, more dispersed national pattern. Two small insets at the top left show outlying islands with sparse clinic distribution.

Navigate back to Figure 1..

